# Dynamic Nonlinear Image Tuning through Magnetic Dipole Quasi‐BIC Ultrathin Resonators

**DOI:** 10.1002/advs.201802119

**Published:** 2019-05-23

**Authors:** Lei Xu, Khosro Zangeneh Kamali, Lujun Huang, Mohsen Rahmani, Alexander Smirnov, Rocio Camacho‐Morales, Yixuan Ma, Guoquan Zhang, Matt Woolley, Dragomir Neshev, Andrey E. Miroshnichenko

**Affiliations:** ^1^ School of Engineering and Information Technology University of New South Wales Canberra ACT 2600 Australia; ^2^ Nonlinear Physics Centre The Australian National University Canberra ACT 2601 Australia; ^3^ Institute of Applied Physics Russian Academy of Sciences Nizhny Novgorod 603950 Russia; ^4^ The MOE Key Laboratory of Weak‐Light Nonlinear Photonics School of Physics and TEDA Applied Physics Institute Nankai University Tianjin 300457 China

**Keywords:** bound state in continuum, dielectric nanostructures, metasurface, nonlinear image tuning, nonlinear nanophotonics

## Abstract

Dynamical tuning of the nonlinear optical wavefront allows for a specific spectral response of predefined profiles, enabling various applications of nonlinear nanophotonics. This study experimentally demonstrates the dynamical switching of images generated by an ultrathin silicon nonlinear metasurface supporting a high‐quality leaky mode, which is formed by partially breaking a bound‐state‐in‐the‐continuum (BIC) generated by the collective magnetic dipole (MD) resonance excited in the subdiffractive periodic systems. Such a quasi‐BIC MD state can be excited directly under normal plane wave incidence and leads to a strong near‐field enhancement to further boost the nonlinear process, resulting in a 500‐fold enhancement of the third‐harmonic emission experimentally. Due to sharp spectral features and asymmetry of the unit cell, it allows for effective tailoring of the nonlinear emissions over spectral or polarization responses. Dynamical nonlinear image tuning is experimentally demonstarted via polarization and wavelength control. The results pave the way for nanophotonics applications such as tunable displays, nonlinear holograms, tunable nanolaser, and ultrathin nonlinear nanodevices with various functionalities.

## 1 Introduction

Aiming for the efficient control of optical properties at the nanoscale, modern nanophotonics focuses on the integration of multiple optical functionalities into single compact on‐chip designs for ultrafast optical switching and optical information processing.^1^, ^2^ Optical nonlinearity plays a critical role in the implementation of such functionalities.^3^ Concentrating light at the subwavelength scale allows one to enhance light–matter interactions and facilitates various nonlinear optical processes, such as frequency conversion, wave mixing, and all‐optical switching.^4^ Engineering nonlinear wavefront enables the formation of nonlinear image generation from nanostructures through either direct image encoding based on the near‐field amplitude^5^ or nonlinear holography method.^6^, ^7^ Nanoplasmonics has been explored extensively in the nonlinear regime due to the strong field confinement arising from the coherent oscillations of conduction electrons near the surface of plasmonic nanostrucutres,^8^, ^9^, ^10^, ^11^, ^12^, ^13^, ^14^, ^15^ however its performance is largely restricted by high Ohmic losses, small mode volumes, and low laser damage threshold.

High‐index dielectric nanostructures have emerged as a promising alternative, and are expected to complement or even replace plasmonic nanostructures for a wide range of potential applications.^16^, ^17^ Besides their complementary metal‐oxide‐semiconductor (CMOS) compatibility and low loss characteristics, dielectric nanostructures offer a powerful platform for efficient manipulation and localization of light based on the control over both optically induced electric and magnetic Mie‐type resonances, offering a great potential for future on‐chip applications in the nonlinear regime.^18^, ^19^ Various dielectric nanostructures supporting the magnetic dipole (MD) resonance,^20^, ^21^, ^22^ nonradiating anapole states,^23^, ^24^ magnetic Fano resonances,^25^ have been explored to confine and manipulate light at the subwavelength scale leading to the enhanced nonlinear response.^26^, ^27^, ^28^, ^29^, ^30^

Fano resonances in photonic periodic systems have been extensively studied and used to achieve strong near‐field enhancement to facilitate light–matter interactions.^31^, ^32^, ^33^, ^34^ The corresponding Fano feature in the optical response is generated through the coupling between two resonances with different damping rates supported by the designed system. A special case of Fano resonances in some designed periodic structures within the light cone can collapse to a bound‐state–in‐the‐continuum (BIC) with the *Q*‐factor going infinity and the disappearance of Fano feature in the spectrum due to the resonance becoming ideally uncoupled from the free‐space radiation.^35^, ^36^

BICs, being originally predicted by von Neumann and Wigner in 1929,^37^ have been extensively studied in different fields of wave physics including acoustics, microwaves, and nanophotonics.^35^, ^38^, ^39^, ^40^, ^41^, ^42^, ^43^, ^44^, ^45^, ^46^, ^47^, ^48^ A special case of symmetry‐protected BICs exist at Γ point of a periodic structure. For example, in a photonic crystal slab, modes above the light line of the band structure are generally radiative due to their coupling to the continuum of the extended modes. However, it is known that some bound state can exist at Γ point even above the light line of the band structure due to symmetry mismatch between their mode profiles and external propagating modes. At Γ point when the operating frequencies are below the diffraction limit, the only radiating states are plane waves in the normal direction with the electromagnetic field being odd under 180° rotation around *z*‐axis, i.e., C_2_ symmetry, thus any even mode at the Γ point is a BIC due to zero overlap between their mode profiles and outgoing waves. In practice, such BICs can be realized as quasi‐BICs by introducing asymmetric factors into the system. The BIC‐induced mechanism of light localization makes it possible to further enhance the performance of Mie‐type resonances in dielectric optically resonant nanostructures, which can be potentially beneficial for linear and nonlinear processes.^49^ Among the multipolar resonances supported by dielectric nanostructures, MD resonance is known for its ability to boost the nonlinear process due to the associated string near‐field enhancement and larger mode volume compared to other multipoles, and thus it has been widely used to enhance the nonlinear frequency conversion.^20^, ^28^

**Figure 1:**
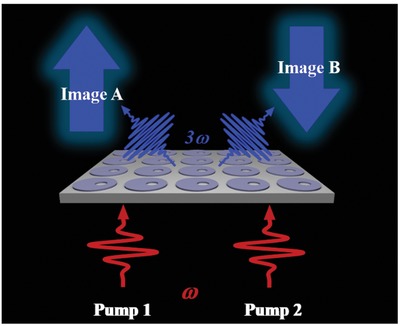
Schematic of third‐harmonic generation and nonlinear image tuning through quasi‐BIC MD resonators. By changing the pump from Pump 1 to Pump 2, the nonlinearly generated image is able to switch from Image A to Image B.

In this work, by taking the advantages of both Mie resonators and BIC states, we design ultrathin resonant silicon disk metasurface supporting high‐quality quasi‐BIC MD state which can be excited directly under normal pump irradiation, leading to a strong near‐field enhancement inside the nanoresonators. We predict theoretically and observe experimentally that such quasi‐BIC MD states can significantly boost the nonlinear response, leading to a total third‐harmonic generation efficiency on the order of 10^−5^ under peak pump intensity value of I0 = 1.0 GW/cm^2^. Owing to the flexible control and tunability over the spectral width and position of such state, we further demonstrate dynamical nonlinear image tuning through designed quasi‐BIC MD resonators, as illustrated in **Figure** **1**. Our results have great potential for perspective nanophotonics applications, such as tunable nanolasers and displays.

## 2 Results and Discussion

An out‐of‐plane MD mode is characterized by the typical in‐plane circulating displacement currents excited inside the nanodisks. The spectral position of the MD mode can be controlled by the changing the aspect‐ratio of the nanodisks. Thus, it is possible to shrink the longitudinal size of the MD resonator to deep‐subwavelength dimensions while maintaining the strong MD resonance. In the following, we focus our working wave‐length in the near‐infrared range (around 1345 nm), and fix the thickness and period of our metasurface to be 53 and 840 nm, respectively.

**Figure 2:**
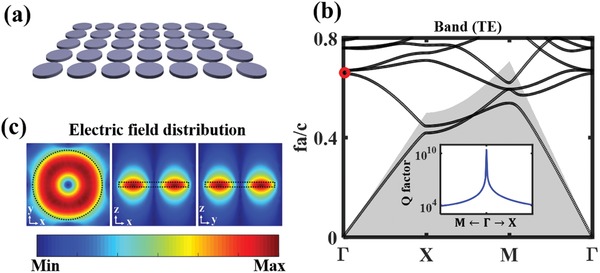
a) Schematic of a metasurface composed of silicon nanodisks. b) Calculated bandgap structure for a periodic 2D array of silicon disks of radius 350 nm. The gray shading indicates the area located below the free‐space light cone. The trapped symmetry‐protected BIC is marked with a red circle. The inset shows the *Q*‐factor of the mode in the 2nd band, which diverges to infinity at Γ point. c) Electric near‐field distributions of the symmetry‐protected BIC.

We first consider a free‐standing metasurface which is composed of a periodic array of silicon nanodisks, as shown in **Figure** **2**a. We perform the band structure calculation based on the Massachusetts Institute of Technology Photonic‐Bands (MPB) open source.^50^ Figure 2b shows the calculated band structure of transverse electric (TE) mode for such ultrathin periodic system. The BIC is formed by the carefully designed nanodisks supporting the resonant normal MD resonance at Γ point with corresponding frequency below the diffraction limit of a given periodic structure. In this case, the only radiating states are plane waves propagating in the normal directions with the electromagnetic field vectors being odd under C_2_ symmetry. The MD mode becomes perfectly confined and does not couple to the free‐space radiation channel any more due to the symmetry mismatch, featuring an infinite *Q*‐factor at Γ point (as shown in the inset of Figure 2b). The *Q*‐factor is estimated by calculating the complex frequency of the modes using the finite element method (FEM) solver through eigenfrequency analysis in COMSOL Multiphysics. The corresponding electric field distributions are shown in Figure 2c. As one can see, the field of designed BIC‐MD state is highly confined inside the nanodisks, being a good candidate for enhancing light–matter interactions and the optical nonlinear processes.

An ideal BIC manifests an infinite *Q*‐factor with complete suppression of the radiation of the state to the free space. By breaking the symmetry of the system which supports a symmetry‐protected BIC, one can transform an ideal BIC to a quai‐BIC, for example, by introducing a defect in the nanodisks, it is possible to open a radiation channel and transform the ideal‐BIC MD state into quasi‐BIC MD state with finite *Q*‐factor. This allows obtaining an energy exchange with the external modes, and manifests itself as a sharp Fano feature in the optical response spectrum. Through the mechanism of BIC, one can simply control and tune the radiation damping rate and thus the width of the resonance (which ideally should vanish when no defect is introduced into the system). So, BIC‐inspired mechanism provides an important and simple tool to engineer and tailor the line width and *Q*‐factor as wanted. Importantly, unlike the conventional confined guided modes supported by the periodic system, which is below the light cone, BIC‐inspired mechanism allows directly exciting the quasi‐BICs by free propagation plane waves, making it a much flexible and novel platform for nanophotonics applications.

**Figure 3:**
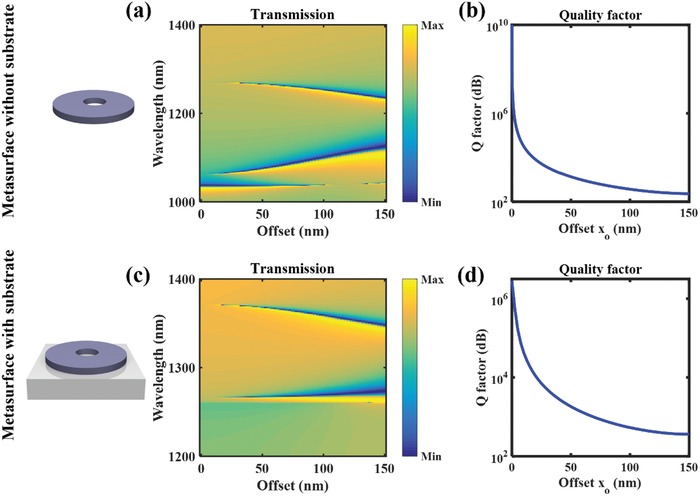
The transmission spectra and *Q*‐factors with respect to the offset of a hole for silicon disk‐hole metasurface in a,b) free space and on c,d) glass substrate, respectively.

Here, we introduce an off‐centered hole to break the C_2_ symmetry and open a leaky channel of the BICs in our system. The typical band structure is similar to Figure 2 (see Figure S1 of the Supporting Information, showing the quasi‐BIC MD state at Γ point). We first calculate the excitation of the quasi‐BIC MD state with different offset *x*_0_ of the hole from the center under normal plane wave incidence. **Figure** **3**a shows the calculated transmission spectra with increasing the offset position of the hole for the nanoresonators placed in free space. As can be seen, the zero offset leads to the ideal BIC MD state with a diverging *Q*‐factor reaching infinity (Figure 3b), and the dip in the transmission vanishes due to absence of the coupling between the input pump and the ideal BIC MD state. A narrow dip emerges from the transmission spectra and becomes broader with increasing the offset value, which corresponds to the excitation of the quasi‐BIC MD state and a decrease of the *Q*‐factor.

For a realistic experimental setup, we further estimate the influence of the glass substrate on such state. Figure 3c,d gives the corresponding results when considering the nanoresonators on a glass substrate. As can be seen, the *Q*‐factor will become smaller due to the substrate, this is due to that the presence of substrate will transform the BIC to a resonant state that leaks into the substrate via radiation channels opening in the substrate.^51^ However, it still maintains high quality and can be easily tuned through the introduced asymmetry factor for desired values.

**Figure 4:**
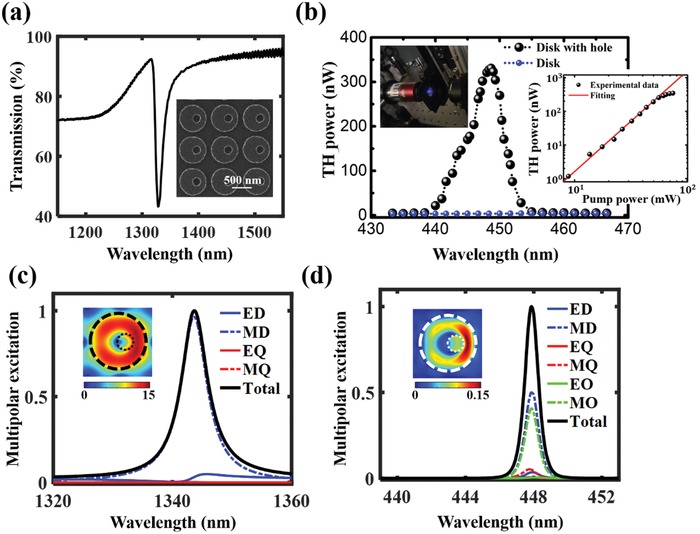
a) Experimentally measured linear transmission spectrum. b) The experimentally measured THG spectra of the sample. The left inset shows a photographic image of the TH emission from the sample, and the right inset shows the measured TH power as a function of pump power, where the cubic dependence is given by the red line *y* = 0.0014*x^3^*. c) Calculated linear multipolar content of the linear light scattering. The inset shows the electric near‐field distribution in *xy* plane at the resonance. d) Calculated multipolar composition of the TH signal. The inset shows the TH near‐field distribution in *xy* plane at the resonance.

Taking into account that the spectral full‐width‐at‐half‐maximum (FWHM) of the experimentally available laser *δλ*_l_ is around 12 nm, we then optimize the quasi‐BIC MD state with its FWHM *δλ*_b_ ≈12 nm to expect the maximum of the nonlinear signal generation (see details in Section II of the Supporting Information). Thus, the silicon metasurface is optimized to support a quasi‐BIC MD state at near‐infrared wavelength around 1345 nm with FWHM around 12 nm. The radii of the nanodisk and hole are *r*_0_ = 350 nm, *r*_h_ = 110 nm, respectively, and the offset *x*_h_ of the hole is 100 nm. Further illustration of the fabrication process of our sample can be found in Section III of the Supporting Information. We first measure the linear transmission spectrum of the metasurface by using plane wave normal incidence, as shown in **Figure** **4**a. A pronounced asymmetric Fano line shape with a narrow dip is observed around λ = 1345 nm, indicating the excitation of the quasi‐BIC‐MD state. The FWHM of the state is estimated experimentally around 12 nm, matching well the available laser width. We further derived the *Q*‐factor from the measured spectrum from the metasurface by fitting the experimentally measured transmission spectrum *T* with a Fano line shape given by $T_{\mathrm{Fano}}=\;\;|a_{1}+ja_{2}+\frac{b}{\omega -\omega_{0}+j\gamma }{|}^{2}$, where *a*_1_, *a*_2_, and *b* are constant real numbers, ω_0_ is the central resonant frequency; γ is the overall damping rate of the resonance. The experimental *Q*‐factor was then estimated as 128 via formula *Q* = ω_0_ /2γ^32^ (with ω_0_ = 1.41 × 10^15^ Hz, and other fitting parameters being *a*_1_ = 0.64, *a*_2_ = 0.78, *b* = 1.9 × 10^12^, and γ = 5.5 × 10^12^ Hz). The finite lateral size of metasurface will influence the *Q*‐factor due to the lattice perturbations at the array's edge breaking the coherence and leading to strong scattering of light into free space, hence broadening the resonance peak.^32^, ^52^, ^53^ By investigating the angular dependence of the resonance, we further estimate that the corresponding coherence length is about *l*_c_ ≈ 20 μm. Thus, such *Q*‐factor can be generally supported when the laterial size of the metasurface is larger than *l*_c_ (see Section IV in the Supporting Information^54^, ^55^).

By performing the multipolar decomposition of the optical linear response, shown in Figure 4c, we find that such state is dominated by the MD response, with smaller contributions from the electric dipole (ED) resonance representing the radiation channel which couples to the outgoing waves in our system. Such MD feature of the designed quasi‐BIC MD state also can be clearly observed from the near‐field distribution as shown in the inset of Figure 4c. We then perform the third‐harmonic spectroscopy measurement (For experimental setup of the nonlinear measurement, see Section V of the Supporting Information). The third‐harmonic generation (THG) was measured from the metasurface at different pump wavelengths. Figure 4b shows the experimentally measured wavelength dependence of the THG emission. For comparison, we also measured the THG wavelength dependence from silicon disk metasurface (without the introduction of an off‐centered hole).

The central wavelength of the pump beam was tuned ranging from 1300 to 1400 nm, with maximum mean power in the sample plane up to around 100 mW. The pump beam with 200 fs pulse width and 80 MHz repetition rate was focused by an aspheric lens with focal‐length being 5 cm to a beam waist of 20 µm leading to a maximum peak intensity value of *I*_0_ = 1.0 GW cm^−2^. We use another objective with NA = 0.7 to collect the transmitted TH emission. As can be seen, the TH signal can be dramatically enhanced in the optimized disk‐hole metasurface at the quasi‐BIC‐MD state (around the linear transmission dip of the Fano line shape), leading to an estimated forward TH conversion efficiency around 5 × 10^−6^. By comparing with the case for solid disk metasurfaces, more than 500 times stronger TH signal is observed. Here, the TH conversion efficiency is limited by the finite size of the metasurfaces. Theoretical analysis shows that only 40% of the total TH emission power goes in the forward direction, therefore the total TH conversion efficiency is estimated in the order of ≈10^−5^ (see Figure S6, Supporting Information). We further analyze the multipolar structure of the nonlinear emission, as shown in Figure 4d, where the inset shows the nonlinear near‐field distribution in the *xy* plane. When the pump wavelength is in the vicinity of the quasi‐BIC MD state, a much stronger nonlinear near‐field distribution can be obtained compared to the case when pump wavelength is off‐resonant (see Figure S7, Supporting Information). In agreement with the multipolar theory described in ref. 56, the TH signal features a magnetic nature with predominant excitation of MD and magnetic octupole resonances, which exhibit the same symmetry with respect to C_2_. Also, there is a small portion of magnetic quadrupole and ED excitation based on the generated nonlinear multipoles. This further leads to a stronger emission in the first‐order diffraction compared to the zero‐order diffraction, due to absence of coupling to the magnetic multipoles and the normal outgoing waves (see Figures S8 and S9 in the Supporting Information). From the inset of Figure 4b, one sees that the nonlinear signal gradually reaches saturation at higher pump power. Such saturation occurs in the fundamental power transmission due to the two‐photon absorption and subsequent free‐carrier absorption effects which will eventually damage the sample.^20^ However, we still achieve a total TH convention efficiency in the order of ≈10^−5^ using pump fluency only *I*_0_ = 1.0 GW cm^−2^. It is expected that this efficiency can be further improved if using a laser with a smaller line width (see Section 2 of the Supporting Information), or by designing doubly high‐*Q* resonances at both fundamental wavelength and harmonic wavelength, such as doubly BIC state, it will further relieve the restriction on the pump power at the fundamental wavelength and thus can dramatically boost the nonlinear process to a further level.

**Figure 5:**
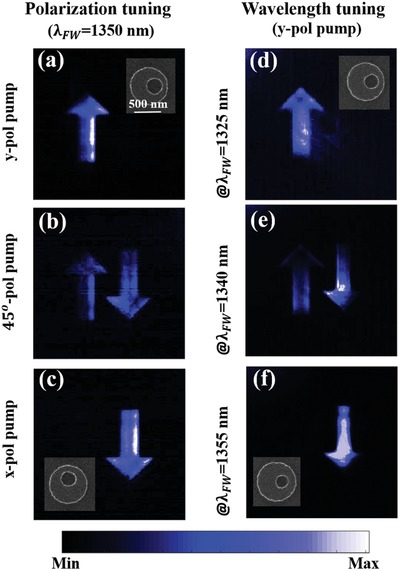
Nonlinear image tuning through the designed metasurfaces by polarization tuning and wavelength tuning respectively. a–c) Under 1350 nm pump incidence with polarization being *y*‐pol, 45°‐pol and *x*‐pol, respectively. Here, *r*_0_ = 356 nm. *r*_h_ = 131 nm. d–f) Under *y*‐pol pump incidence with wavelength at 1325, 1340, and 1355 nm, respectively. *r*_0_ = 344 nm, *r*_h_ = 122 nm for ↑ and *r*_0_ = 356 nm, *r*_h_ = 110 nm for ↓.

Such high‐*Q* quasi‐BIC MD states provide a powerful platform to spatially control the nonlinear emission efficiently and dynamically. In the following, we demonstrate two simple methods to show the dynamical nonlinear image tuning. According to Figure 4, the quasi‐BIC MD state can dramatically enhance the nonlinear emission from the nanostructures, thus if we encode two images into the metasurface, i.e., using resonances at different wavelength position or under different pump excitation polarization conditions, we expect the image can be tuned to be visible through the THG process via wavelength or polarization tuning. In the following we take the image arrow ↑ as an example to demonstrate this potential. The width and length of arrow are about 20 and 90 µm, respectively. Due to spatial asymmetry of such quasi‐BIC‐MD states, we first encode two images of arrow ↑ and ↓ indicating the opposite directions through the metasurface for disks with *x*‐off‐centered hole and disk with *y*‐off‐centered hole, respectively. By varying the pump from *x*‐polarization to *y*‐polarization, it is possible to separately excite the quasi‐BIC MD states supported by the two images. Thus, one can readout the encoded image through the generated nonlinear signal. The measured nonlinear images for *x*‐polarized, 45°‐polarized and *y*‐polarized pump, respectively, are shown in **Figure** **5**a–c. As the spectral position and width of such quasi‐BIC MD state can be engineered effectively through geometric tuning of the nanoresonators, the dynamical nonlinear image tuning can also be achieved directly through wavelength tuning, as shown in Figure 5d–f where the two images are encoded into the nanoresonators with quasi‐BIC MD state at different spectral positions (at 1325 and 1355 nm). It is worth mentioning that, owing to the sharp high‐quality quasi‐BIC MD state, reversible thermal tuning of the nonlinear emission and nonlinear image switching are also possible based on the large thermo‐optical effect of silicon.^57^

In summary, we have demonstrated an ultrathin resonant silicon metasurface supporting hiqh‐*Q* quasi‐BIC MD state. Due to strong light confinement inside the nanoresonators originating from the BIC‐MD nature, we have shown that such quasi‐BIC MD state can significantly enhance the nonlinear process, leading to a total TH conversion efficiency on the order of 10^−5^ under peak pump intensity *I*_0_ = 1.0 GW cm^−2^. Furthermore, such high‐*Q* quasi‐BIC MD states allow for active control on the nonlinear emission. Finally, we demonstrated a dynamical nonlinear image switching through polarization‐ and wavelength‐tuning methods, respectively. Our results offer unique opportunities for constructing nonlinear optical effects to achieve novel nonlinear photonic metadevices with multifunctionalities, such as nonlinear tunable displays, nanolasers, etc.

## Conflict of Interest

The authors declare no conflict of interest.

## Supporting information

Supplementary
