# Supplementary material for: Dynamic Nonlinear Image Tuning through Magnetic Dipole Quasi‐BIC Ultrathin Resonators
Source: Adv Sci (Weinh). 2019 May 23;6(15):1802119. doi: 10.1002/advs.201802119 (PMC6685498; doi:10.1002/advs.201802119)
Supplement: Supplementary file 1 — Supplementary [file ADVS-6-1802119-s001.pdf]

Copyright WILEY-VCH Verlag GmbH & Co. KGaA, 69469 Weinheim, Germany, 2019.

Supporting Information

for *Adv. Sci.,* DOI: 10.1002/advs.201802119

Dynamic Nonlinear Image Tuning through Magnetic Dipole Quasi-BIC Ultrathin Resonators

*Lei Xu, Khosro Zangeneh Kamali, Lujun Huang, Mohsen Rahmani, Alexander Smirnov, Rocio Camacho-Morales, Yixuan Ma, Guoquan Zhang, Matt Woolley, Dragomir Neshev, and Andrey E. Miroshnichenko**

Copyright WILEY-VCH Verlag GmbH & Co. KGaA, 69469 Weinheim, Germany, 2019.

Supporting Information

Dynamic nonlinear image tuning through magnetic dipole quasi-BIC ultra-thin resonators

*Lei Xu, Khosro Zangeneh Kamali, Lujun Huang, Mohsen Rahmani, Alexander Smirnov, Rocio Camacho-Morales, Yixuan Ma, Guoquan Zhang, Matt Woolley, Dragomir Neshev, and Andrey E. Miroshnichenko**

Dr. Lei Xu, Dr. Lujun Huang, Dr. Matt Woolley, Prof. Andrey E. Miroshnichenko
School of Engineering and Information Technology, University of New South Wales, Canberra ACT 2600, Australia
E-mail: andrey.miroshnichenko@unsw.edu.au

Mr. Khosro Zangeneh Kamali, Dr. Mohsen Rahmani, Ms. Rocio Camacho-Morales, Prof. Dragomir Neshev
Nonlinear Physics Centre, The Australian National University, Canberra ACT 2601, Australia

Dr. Alexander Smirnov
Institute of Applied Physics, Russian Academy of Sciences, Nizhny Novgorod 603950, Russia

Mr. Yixuan Ma, Prof. Guoquan Zhang
The MOE Key Laboratory of Weak-Light Nonlinear Photonics, School of Physics and TEDA Applied Physics Institute, Nankai University, Tianjin 300457, China

Contents

I. **Linear optical calculation**

II. **Quasi-BIC MD state optimization**

III. **Nanofabrication**

IV. **Q-factor and coherent length extraction for the quasi-BIC MD state**

V. **Experimental setup for nonlinear measurements**

VI. **Third-Harmonic Generation Calculation**

I. Quasi-BIC MD state optimization

Figure S2 shows the calculated band structure for the periodic nanosystem composed by disk-hole quasi-BIC-MD resonators in free space. By introducing proper asymmetry factor into such system, we transformed the BIC MD state which has infinite Q-factor to quasi-BIC MD state with specific finite Q-factor.

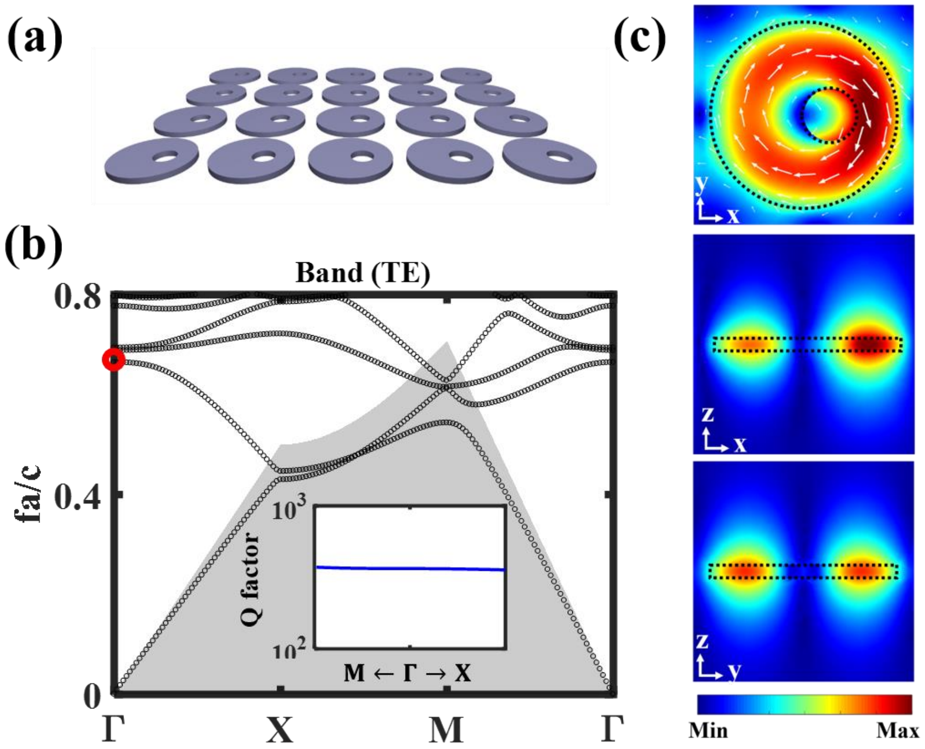

Figure S1. (a) Schematic of silicon disk-hole metasurface. Here, the radius of disk and off-centered hole is $r = 350\ nm$, $r_h = 110\ nm$ with offset $x_h = 100\ nm$. (b) Calculated band structure. The gray shaded area indicates the light cone of the free space. The inset shows the Q-factor evolution of the mode in the 2nd band near Γ point. (c) Electric near-field distributions of the quasi-BIC MD state at Γ point.

II. Quasi-BIC MD state optimization

Considering a pump laser with total intensity I_0 at wavelength λ with spectral width $\delta\lambda_l$. The intensity at each wavelength point within the spectral range $[-\delta\lambda_l/2, \delta\lambda_l/2]$ can be estimated as $I_{inc} = \frac{I_0}{\delta\lambda_l}$.

For a designed quasi-BIC MD state with mode width $\delta\lambda_b$, the corresponding Q-factor can be evaluated by

$$Q = \frac{f_r}{\delta f} = \frac{\omega_r}{\delta\omega} = \frac{\lambda}{\delta\lambda}$$

Or estimated by the energy stored inside the resonator as

$$Q = 2\pi \frac{(energy\ stored)}{(energy\ dissipated\ per\ cycle)}$$

If the intensity enhancement inside the resonator is $I = nI_{inc}$. Then we can get the following relation

$$Q \sim 2\pi \frac{nI\_inc}{I\_inc} = 2\pi n$$

During the nonlinear process, take third-harmonic generation process as an example, the nonlinear emission intensity is cubic dependent on the pump intensity.

The total nonlinear emission power

$$I_{nl} \sim A \int_{-\delta\lambda_l/2}^{\delta\lambda_l/2} I^3 d\lambda = A \int_{-\delta\lambda_l/2}^{\delta\lambda_l/2} (nI_{inc})^3 d\lambda = A \int_{-\delta\lambda_l/2}^{\delta\lambda_l/2} (\frac{\lambda}{\delta\lambda_b})^3 {I_{inc}}^3 d\lambda = \frac{A}{{\delta\lambda_b}^3} \int_{-\delta\lambda_l/2}^{\delta\lambda_l/2} \lambda^3 {I_{inc}}^3 d\lambda$$

Considering that the total input pump is constant, the intensity at each wavelength can be written as $I_{inc} \sim \frac{P}{\delta\lambda_l}$

when $\delta\lambda_b \leq \delta\lambda_l$, the total nonlinear emission can be estimate roughly as

$$I_{nl} \sim \frac{A}{{\delta\lambda_b}^3} \int_{-\delta\lambda_b/2}^{\delta b/2} \lambda^3 {I_{inc}}^3 d\lambda \sim \frac{1}{4} \delta\lambda_b \frac{P^3}{{\delta\lambda_l}^3}$$

when $\delta\lambda_b \geq \delta\lambda_l$, the total nonlinear emission can be estimate roughly as

$$I_{nl} \sim \frac{A}{{\delta\lambda_b}^3} \int_{-\delta\lambda_l/2}^{\delta l/2} \lambda^3 {I_{inc}}^3 d\lambda \sim \frac{1}{4} \delta\lambda_l \frac{P^3}{{\delta\lambda_b}^3}$$

Thus, for a given laser width $\delta\lambda_l$, in order to obtain large total nonlinear emission power, the optimized width for the mode $\delta\lambda_b$ is equal to $\delta\lambda_l$. Experimentally, our laser has a spectral width around 12 nm, so we choose the parameters of our quasi-BIC MD resonator as follows to achieve a comparable width: $\mathrm{r} = 350$ nm, $\mathrm{r_h} = 110$ nm, $\mathrm{x_h} = 100$ nm.

III. Nanofabrication

The fabrication of the metasurface was started by depositing a 53nm-thick layer of amorphous Silicon (*a*-Si) on a glass substrate at 250°C through plasma-enhanced chemical vapor deposition (PECVD) process. Before patterning, the deposited film went through a thermal treatment at 400˚C for 5 minutes. The Electron-beam lithography was on the a-Si film by using a positive-tone (ZEP520A) electroresist, followed by evaporation of a thin layer of chromium to generate metallic masks. Subsequently, patterns were transferred to the a-Si film by induction coupled plasma (ICP) etching. Finally, the metallic masks were removed by wet etching. Figure S1 gives the schematic illustration of the fabrication process for our sample.

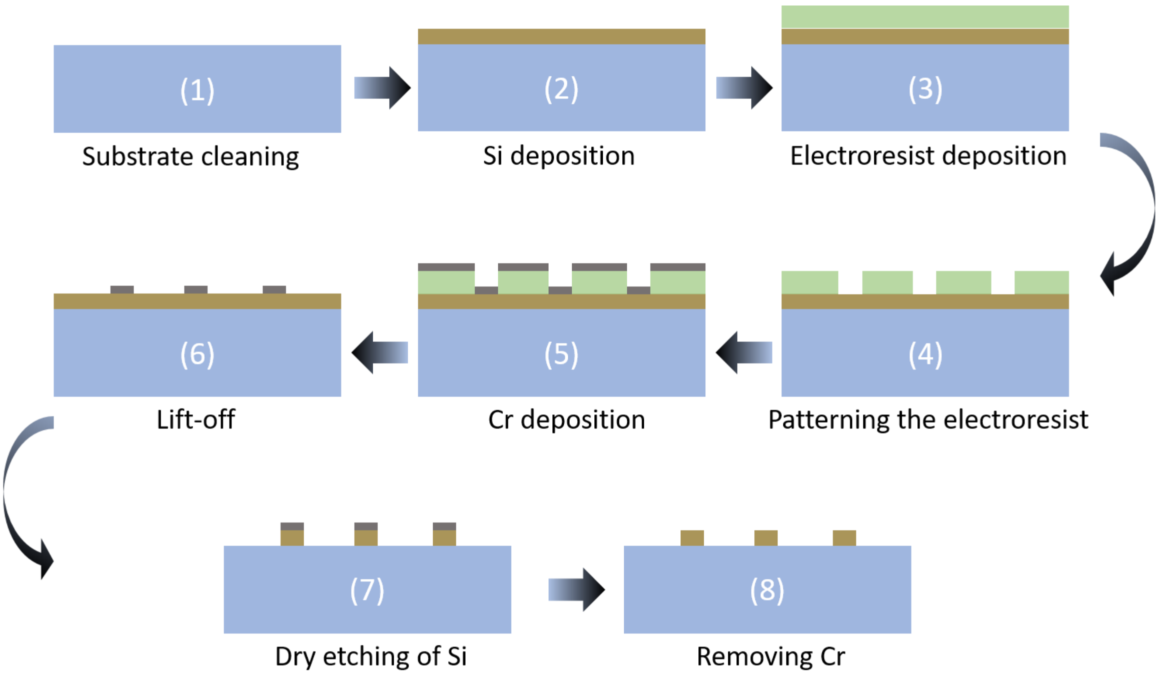

Figure S2. Illustration of the nanofabrication steps.

IV. Q-factor and coherent length extraction for the quasi-BIC MD state

We derived the Q-factor from the measured spectrum from the metasurface by fitting the experimentally measured transmission spectrum T with a Fano line shape given by $T_{Fano} = \left|a_1 + ja_2 + \frac{b}{\omega - \omega_{0+j\gamma}}\right|^2$ [2], where a_1, a_2 and b are constant real numbers, ω_0 is the central resonant frequency; γ is the overall damping rate of the resonance. The experimental Q-factor was then estimated as 128 via formula $Q = \omega_0/2\gamma$ (with $\omega_0 = 1.41 \times 10^{15}\text{Hz}$, and other fitting parameters being $a_1 = 0.64$, $a_2 = 0.78$, $b = 1.9 \times 10^{12}$, and $\gamma = 5.5 \times 10^{12}\text{Hz}$).

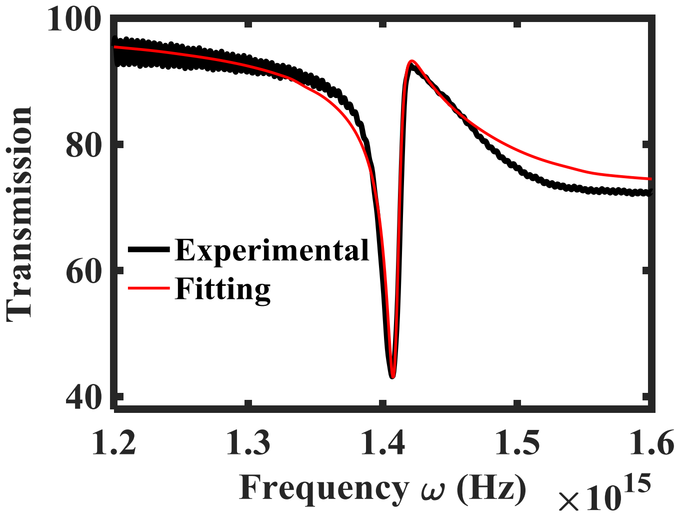

Figure S3. The experimentally measured transmission spectrum fitted with a Fano line shape.

By investigating the angular dependence of the resonance, we further estimate the coherent length l_c via $l_c \approx \lambda/\Delta\theta \approx 20\mu m$ [3,4].

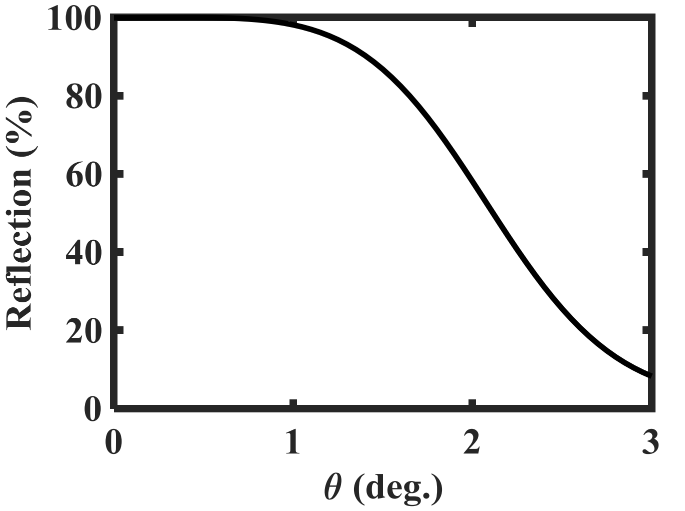

Figure S4. The reflection spectrum at the resonance position as a function of the incident angle θ.

V. Experimental setup for nonlinear measurements

The schematic of the experimental setup for the nonlinear measurements is shown below. Objectives with ×100 NA0.7 and ×20 NA0.4 are used for THG spectroscopy measurement and THG image measurement, respectively.

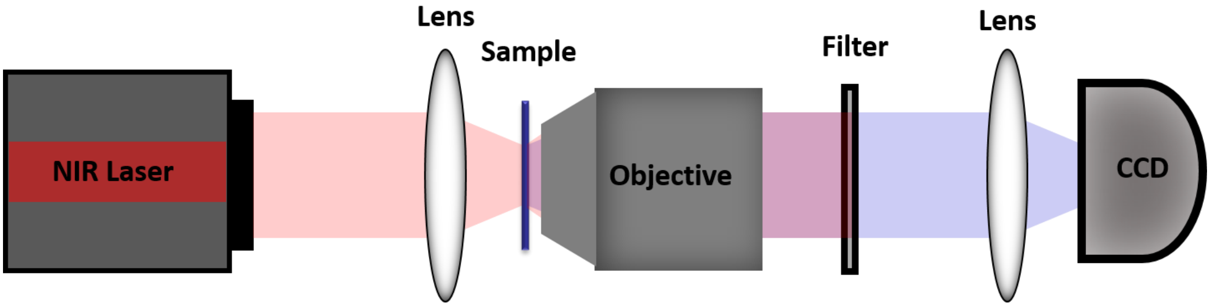

Figure S5. Schematic of the experimental setup for the nonlinear measurements.

VI. Third-Harmonic Generation Calculation

The nonlinear response of our structure is modeled by use of FEM solver in COMSOL Multiphysics in frequency domain. We assume undepleted pump field and follow two coupled steps to emulate the nonlinear THG process: First, we model the linear scattering at the fundamental frequency under plane wave normal excitation. We neglect surface nonlinearities due to their negligible contribution to the THG process for centrosymmetric materials such as silicon [1]. The nonlinear susceptibility tensor $\chi^{(3)}$ is considered as a constant scalar value for amorphous silicon material, and the induced nonlinear polarization components at the third harmonic wavelength can be simplified as $P_i^{(3)} = \varepsilon_0 \chi^{(3)} \mathrm{E}_i(\mathbf{E} \cdot \mathbf{E})$ where i refers to the components in x, y, z directions, respectively. Next, we employ the bulk nonlinear polarization induced inside the nanoparticles as a source to perform the electromagnetic simulation at the harmonic frequency to obtain the generated TH field. We calculate the proportion of the forward and backward THG emission as shown in Figure S5. As can be seen, around 40% of the total THG emission goes in the forward direction.

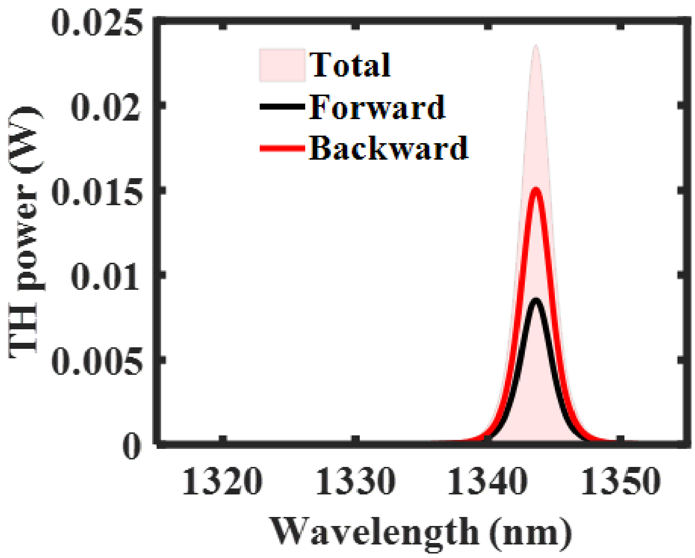

Figure S6. Calculated total TH emission power from quasi-BIC MD resonators.

We further calculate the TH near-field distributions when pump at the resonance and off resonance, as shown in Figure S6.

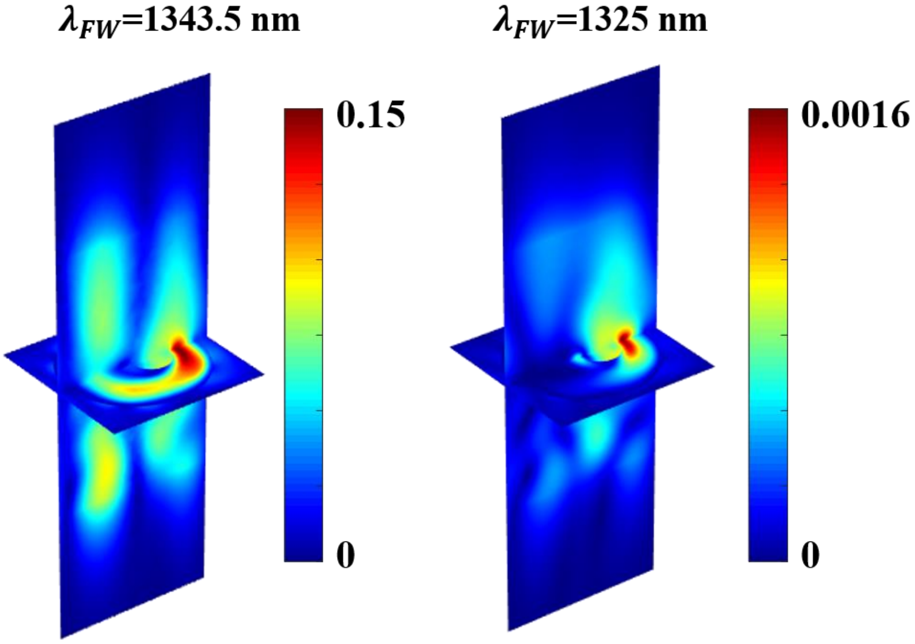

Figure S7. Electric near-field distributions of the harmonic waves for pump wavelength being 1343.5 nm (at the quasi-BIC MD state) and 1325 nm (off resonance), respectively.

By performing the Fourier transform of the THG field in both the forward and backward directions, we obtain the corresponding THG diffractions, as shown in Figure S7. As can be seen, the nonlinear emission mainly goes into the first-order diffractions, with only 4% (2%) of the emission goes in the forward (backward) zero-order directions. This can be explained well based on the magnetic nature of nonlinearly-generated multipoles, i.e., magnetic multipoles cannot couple to the normal outgoing waves due to the zero overlap between them and the radiative outgoing waves.

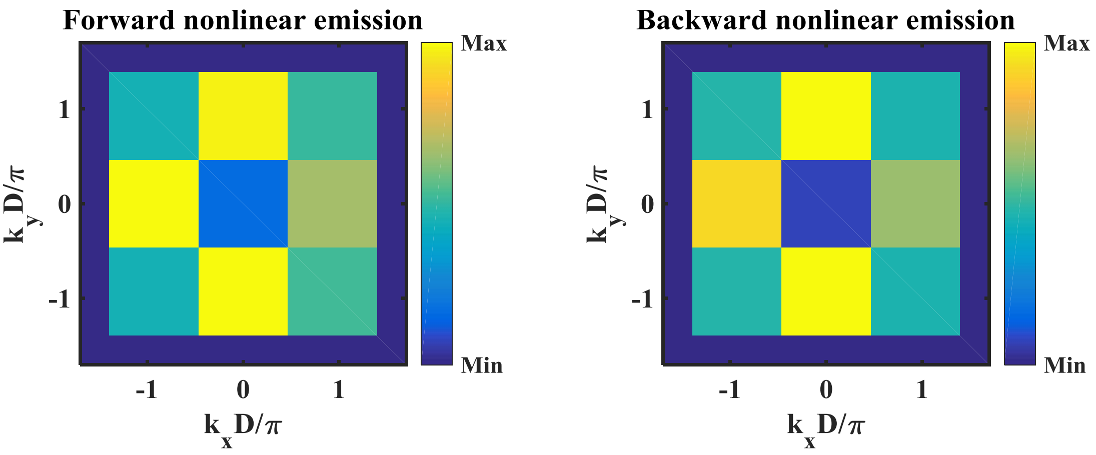

Figure S8. Forward and backward far-field diffractions of the harmonic waves for pump wavelength being 1343.5 nm at the quasi-BIC MD state.

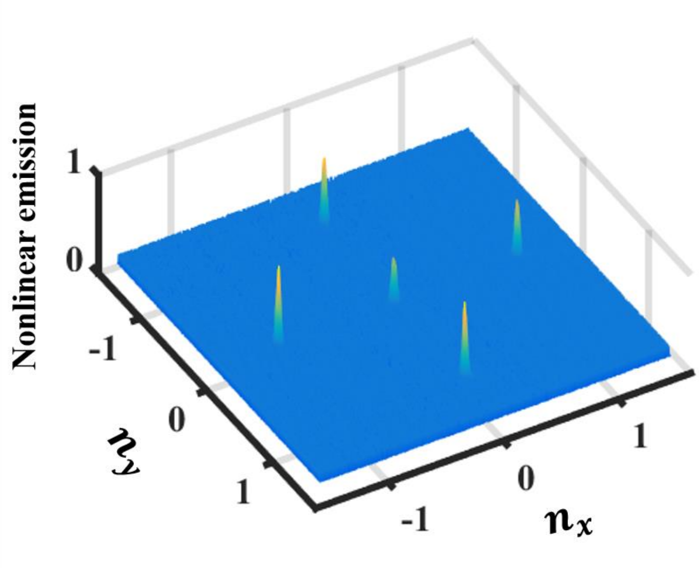

Figure S9. Experimentally measured forward far-field diffraction of the TH signal for pump wavelength being 1343.5 nm at the quasi-BIC MD state.

References

[1] J. E. Sipe, D. J. Moss, H. M. Van Driel, "Phenomenological theory of optical second- and third-harmonic generation from cubic centrosymmetric crystals," Phys. Rev. B: Condens. Matter 35, 1129 (1987).

[2] Y. Yang, I. I. Kravchenko, D. P. Briggs, and J. Valentine, "All-dielectric metasurface analogue of electromagnetically induced transparency," Nat. communications 5, 5753 (2014).

[3] J.-J. Greffet, R. Carminati, K. Joulain, J.-P. Mulet, S. Mainguy, and Y. Chen, "Coherent emission of light by thermal sources," Nature 416, 61 (2002).

[4] Z. Wei, H. Li, Y. Cao, C. Wu, J. Ren, Z. Hang, H. Chen, D. Zhang, and C. Chan, "Spatially coherent surface resonance states derived from magnetic resonances," New J. Phys. 12, 093020 (2010).
